# Direct numerical simulations of three-dimensional surface instability patterns in thin film-compliant substrate structures

**DOI:** 10.1038/s41598-021-95414-8

**Published:** 2021-08-12

**Authors:** Siavash Nikravesh, Donghyeon Ryu, Yu-Lin Shen

**Affiliations:** 1 Department of Mechanical Engineering, University of New Mexico, Albuquerque, NM 87131 USA; 2 Department of Mechanical Engineering, New Mexico Institute of Mining and Technology, Socorro, NM 87801 USA

**Keywords:** Engineering, Materials science

## Abstract

A comprehensive numerical study of three-dimensional surface instability patterns is presented. The formation of wrinkles is a consequence of deformation instability when a thin film, bonded to a compliant substrate, is subject to in-plane compressive loading. We apply a recently developed computational approach to directly simulate complex surface wrinkling from pre-instability to post-instability in a straightforward manner, covering the entire biaxial loading spectrum from pure uniaxial to pure equi-biaxial compression. The simulations use embedded imperfections with perturbed material properties at the film-substrate interface. This approach not only triggers the first bifurcation mode but also activates subsequent post-buckling states, thus capable of predicting the temporal evolution of wrinkle patterns in one simulation run. The state of biaxiality is found to influence the surface pattern significantly, and each bifurcation mode can be traced back to certain abrupt changes in the overall load–displacement response. Our systematic study reveals how the loading condition dictates the formation of various instability modes including one-dimensional (1D) sinusoidal wrinkles, herringbone, labyrinth, and checkerboard.

## Introduction

When a thin film is bonded to a thick compliant substrate, parallel or more complex forms of wrinkles may develop if the thin film is under compression beyond a critical level. It is a form of mechanical instability, and has received significant attention due to its ubiquity in nature as well as in many modern flexible devices in use or under development. Based on experimental observations various surface wrinkling patterns induced by mechanical load, temperature and structural change have been recognized^1–12^. Analytical treatments have been reported addressing the simple one-dimensional (1D) sinusoidal wrinkle pattern^2,13–16^ and the more complex two-dimensional (2D) surface patterns^17–23^. Computational modeling of wrinkle formation using the finite element technique is laborious. A common practice is to undertake a pre-instability linear modal analysis, followed by a separate post-instability analysis involving perturbations in geometry, boundary condition or mesh^15,24–28^. In these approaches, the simulation process frequently involved prescribing a small wave pattern in the model, thus actually dictating subsequent evolution of wrinkle morphologies. Other numerical schemes such as those based on the applied dummy/fictitious load have been developed^29–32^, which are still a multi-step process. Dynamic numerical analyses have also been employed^33,34^, but verification of the numerical results may be difficult due to the lack of reliable closed-form solutions to surface instability problems with the inertial effect. In this work we employ a recently developed numerical approach to directly simulate complex surface wrinkling from pre-instability to post-instability in a seamless manner, covering the entire biaxial loading spectrum from pure uniaxial compression to pure equi-biaxial compression. The numerical predictions also provide mechanistic rationale for uncertainties seen from past theoretical and experimental considerations.

The simulations are accomplished by incorporating pre-existing material defects in the numerical model. Embedded imperfections are regular finite elements at the film-substrate interface but with perturbed material properties, to trigger the instability states. In this study we show that the embedded imperfections not only can initiate the first bifurcation mode, but can also activate any subsequent post-bifurcation instability states and therefore lead to continuous evolution of surface instability patterns. Surface wrinkling can be captured without the need of any tedious or multi-step numerical treatments. This approach was first developed for 1D sinusoidal wrinkling simulations^35–37^, and was recently extended to simple forms of 3D surface wrinkles^38^. The current paper presents our first comprehensive study on surface patterns encompassing the full range of in-plane compressive loading. Instead of treating surface patterns discretely under specific assumptions, we aim to provide a full picture by applying our numerical approach to directly simulate any possible forms of wrinkling instabilities and their transition.

It is worth mentioning that the terms “direct numerical simulations” and “instability” used in this paper should not be mistaken as similar terminologies in the fluid mechanics literature. Here the use of direct numerical simulations is to distinguish between the common multi-step approaches in the solid mechanics literature and our embedded imperfection technique which only requires a single-step analysis. Furthermore, instability in solid structures under compression is frequently referred to as the onset of buckling, as opposed to its definition in fluid mechanics as the onset of development of turbulence. Nevertheless, apparent similarities exist and one may draw analogies between the present surface instability analysis in solid materials and the studies of receptivity^39^ or non-modal disturbance growth^40^ in fluid mechanics.

## Brief overview of theories

Various theoretical formulations of surface instability are available in the literature. Here we include representative analytical solutions, some of which are used for numerical model verifications in the current study. Detailed discussion of the theories is given in the Supplementary Information of this paper. For surface wrinkling of a thin film on top of a compliant substrate, one may categorize available analytical techniques into generic groups of 1D^2,13–16^ and 2D buckling formulations^17–23,41^. The 1D solutions, based on the plane strain assumption, focus on the classical sinusoidal wrinkles (also termed 1D wrinkles). The 2D analytical solutions allow for in-plane biaxial loading.

**Figure 1:**
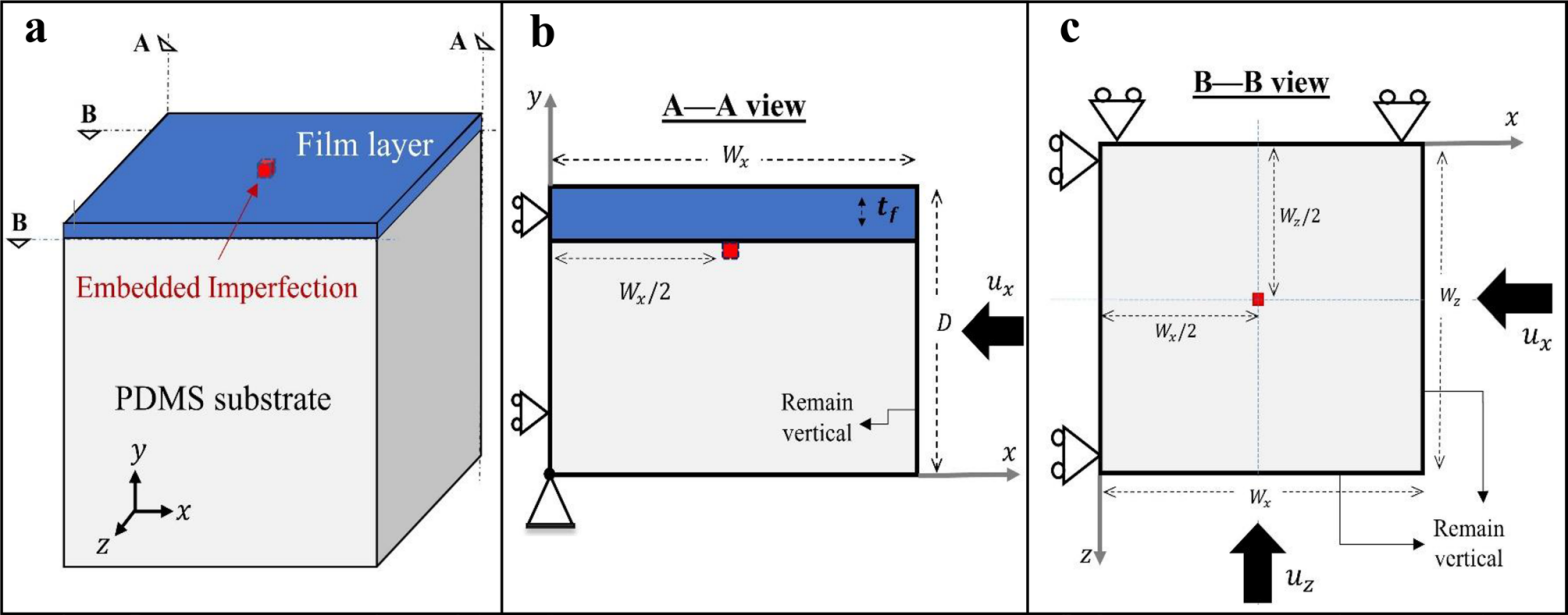
(**a**–**c**) Schematics of the problem domain, boundary conditions, and the directions of applied displacement.

Consider the thin film-substrate structure shown in Fig. 1. Under uniaxial compression, 1D sinusoidal wrinkling commences once the critical point for instability is reached. Assuming that the substrate is semi-infinite, film and substrate are fully bonded at the interface, and both layers are linear-elastic and isotropic, the wavelength of the wrinkles at the onset of bifurcation (primary instability) follows

1 $$\left( {\lambda_{cr} } \right)_{1D} = \lambda_{cr} = 2\pi t_{f} \left[ {\frac{{E_{f} }}{{3\left( {1 - \upsilon_{f}^{2} } \right)\overline{E}_{s} }}} \right]^{1/3} ,$$

where $\lambda_{cr}$ is the critical wavelength of the 1D mode, and $t_{f} ,$ $E_{f}$, and $\nu_{f}$ are, respectively, thickness, Young’s modulus, and Poisson’s ratio of the film layer. The parameter $\overline{E}_{s}$ = $E_{s} /\left( {1 - \upsilon_{s}^{2} } \right)$, where $\nu_{s}$ and $E_{s}$ are Poisson’s ratio and Young’s modulus, respectively, of the substrate. An alternative expression for the parameter $\overline{E}_{s}$ is given^17,19,23^ as $\overline{E}_{s}$ = $\left[ {E_{s} /\left( {1 - \upsilon_{s}^{2} } \right)} \right] \cdot \left[ {4\left( {1 - \upsilon_{s} } \right)^{2} /\left( {3 - 4\upsilon_{s} } \right)} \right]$. However, the difference between the two forms of $\overline{E}_{s}$ is negligible for near-incompressible cases; for a fully-incompressible substrate ($\upsilon_{s}$ = 0.5), $\left[ {4\left( {1 - \upsilon_{s} } \right)^{2} /\left( {3 - 4\upsilon_{s} } \right)} \right] = 1$.

The critical wrinkling stress $, {\sigma }_{cr}$, corresponding to ${\lambda }_{cr}$ was also derived as^13,18,42^

2 $$\sigma_{cr} = \left[ {\frac{{E_{f} }}{{4\left( {1 - \upsilon_{f}^{2} } \right)}}} \right]\left[ {\frac{{3\left( {1 - \upsilon_{f}^{2} } \right)\overline{E}_{s} }}{{E_{f} }}} \right]^{2/3} ,$$

assuming that the stress state is uniform in the film layer (with the cross-section area of $t_{f} \cdot w_{z}$ as shown in Fig. 1). The critical strain for 1D wrinkling, $e_{cr}$, was reported as^13,18,23^,

3 $$\left( {e_{cr} } \right)_{1D} = e_{cr} = \left( \frac{1}{4} \right)\left[ {\frac{{3\left( {1 - \upsilon_{f}^{2} } \right)\overline{E}_{s} }}{{E_{f} }}} \right]^{2/3} .$$

Note that it is equivalent to the critical stress in Eq. (2) divided by the plane-strain modulus of the film, $E_{f} /\left( {1 - \upsilon_{f}^{2} } \right)$. For a film-substrate structure subjected to pure equi-biaxial compression, the square-checkerboard pattern has been shown to possess the lowest energy in the buckled state^17,19^. The wavelength of square-checkerboard was derived^17,18,20^ as

4 $$\left( {\lambda_{cr} } \right)_{Cb} = \sqrt {2 } \left( {\lambda_{cr} } \right)_{1D} = 2\sqrt 2 \pi t_{f} \left[ {\frac{{E_{f} }}{{3\left( {1 - \upsilon_{f}^{2} } \right)\overline{E}_{s} }}} \right]^{1/3} .$$

It was also postulated that the critical stress introduced in Eq. (2) not only applies to 1D wrinkles but also applies to any possible biaxial wrinkling mode. The critical strain for the square-checkerboard mode, $\left( {e_{cr} } \right)_{CB}$, is apparently derived^17,18,20^ by dividing $\sigma_{cr}$ by the biaxial modulus of the film, $E_{f} /\left( {1 - \upsilon_{f} } \right)$,

5 $$\left( {e_{cr} } \right)_{CB} = \frac{{\sigma_{cr} }}{{\left[ {E_{f} /\left( {1 - \upsilon_{f} } \right)} \right]}} = \left[ {\frac{1}{{4\left( {1 + \upsilon_{f} } \right)}}} \right]\left[ {\frac{{3\left( {1 - \upsilon_{f}^{2} } \right)\overline{E}_{s} }}{{E_{f} }}} \right]^{2/3} .$$

In addition, the amplitude of the surface wrinkles, $A$, can be written in a general form as

6 $$A = {\Psi }\left( {\frac{e}{{e_{cr} }} - 1} \right)^{1/2} ,$$

where the parameter ${\Psi }$ is a function of Poisson’s ratio and thickness of the film layer, with ${\Psi }$ = $t_{f}$ for the sinusoidal 1D mode and ${\Psi }$ = $t_{f} \cdot \sqrt {8/[\left( {3 - \upsilon_{f} } \right)\left( {1 + \upsilon_{f} } \right)]}$ for the square-checkerboard mode^17,20^, and $e/e_{cr}$ is the applied compressive strain normalized by the critical value at the onset of primary bifurcation.

Controversies exist in the literature regarding the wrinkling patterns pertaining to specific loading states. There are other well recognized surface instability patterns such as herringbone (or zigzag) and labyrinth, which emerge after the primary modes when the compressive stress is well beyond the first critical point. There are also uncertainties regarding their evolution and necessary loading condition in the post-instability regime. A detailed discussion is given in the Supplementary Information. On the experimental side, although the most energetically favorable mode under equi-biaxial loading is square-checkerboard^17,19^, other checkerboard patterns (including the hexagonal and triangular modes) have been observed in actual experiments^17^. A true square-checkerboard pattern can only form under pre-defined conditions using special fabrication methods^43,44^. As a consequence, researchers have speculated the possible causes of the discrepancy. A pre-existing curvature of the film surface has been considered as a possible cause to initiate hexagonal-mode wrinkles^17,22^. Nonlinear elastic property of the substrate^17^ and unequal substrate elastic moduli in tension and compression have also been proposed as the origin for the hexagon-based checkerboard patterns^21^.

It is evident that, even with a flat film-substrate system with a simple elastic behavior, a unified theme for the evolution of wrinkle patterns is lacking. Furthermore, how transitions occur from one post-instability mode to another is not at all clear. The effects of loading biaxiality are also in need of investigating. In addition to demonstrating the modeling capabilities using the embedded imperfection approach, the present work seeks to address all these issues and offer an overarching scheme of surface wrinkling under biaxial loading.

## Numerical model description

The overall problem geometry and boundary conditions are schematically shown in Fig. 1. A thin-film layer, with thickness $t_{f} = 0.1\,\upmu {\text{m}}$, is on top of a compliant substrate. Both the film and substrate materials are taken to be isotropic linear elastic in all simulations. In this study we consider two film materials separately: P3HT:PCBM (poly-3-hexylthiophene conjugated polymer and phenyl-C61-butyric acid methyl ester fullerene derivative) and PEDOT:PSS (poly-3,4-ethylenedioxythiophene and polystyrene sulfonate acid). These are common polymeric thin films used in organic optoelectronic devices. For the P3HT:PCBM film the elastic modulus is $E_{f} = 7300{ }$ MPa^9^; and for the PEDOT:PSS film it is $E_{f} = 2000{ }$ MPa^45^. This large difference in stiffness helps establish the generality of the current numerical predictions. The Poisson’s ratio is taken as $\nu_{f} = 0.35$ for both film materials. The substrate material is PDMS (polydimethylsiloxane), with elastic modulus of $E_{s} = 2.97{ }$ MPa^46^ and Poisson’s ratio of $\nu_{s} = 0.495$ (set slightly smaller than 0.5 to avoid potential convergence issues).

The simulation domain is defined in such a way to represent a unit cell of a periodic structure. This periodic-cell approach is built upon our earlier 1D wrinkling study^37^ and is now extended to full 3D simulations. One embedded imperfection is placed in the substrate adjacent to the film-substrate interface, as shown in Fig. 1. The pre-existing defect is a regular finite element carrying the elastic property of the film material instead of that of the substrate. To preserve symmetry, the imperfection is of the square shape and exactly at the center of the *xz*-plane (note that all the elements in the model are initially perfectly square shaped in the *xz*-plane). Following the same approach in our two-dimensional simulations^37^, the size of the model is scaled by the analytical critical wavelength of 1D wrinkles ($\lambda_{cr}$ as defined in Eq. 1). The domain dimensions of $W_{x} = W_{z} = 10\lambda_{cr}$ and overall depth *D* = $5\lambda_{cr}$ were chosen. The film surface is thus of square shape, and the substrate is sufficiently thick compared to film thickness. As a consequence of such scaling, the domain dimensions for the cases with P3HT:PCBM film and PEDOT:PSS film were not identical. Nevertheless, this approach is more advantageous since it provides better control over the total number of elements and leads to computationally efficient simulations with straightforward comparisons. It should be noted that the domain dimensions of $W_{x} = W_{z} = 10\lambda_{cr}$ were intentionally chosen such that $10\lambda_{cr} \cong 7\left( {\lambda_{cr} } \right)_{Cb}$, meaning that the model size is also sufficiently large for the formation of 7 cycles of square-checkerboard waves (based on Eq. 4) in each of the *x* and *z* directions. In addition, the model size is presumably suitable for the post-buckling studies of herringbone and labyrinth patterns, following the rationale discussed in the Supplementary Information that the short wavelength of the surface wrinkling patterns remains invariant and identical to the wavelength of 1D mode in post-instability regimes. Therefore, the model size is appropriate for capturing a variety of wrinkling features, and is also suitable for verification studies by comparing the results with available analytical solutions.

It is noted that, since the embedded imperfection is a regular finite element, the imperfection size will be affected as the mesh density is altered. From our previous studies, the simulated surface wrinkling features were found to be essentially independent of the imperfection size^37,38^ and material property^36^ as long as mesh convergence is achieved. It was also shown that the out-of-plane imperfection thickness of 0.5 $t_{f}$, used also in the current study, has a negligible effect on the numerical prediction^38^. In addition, it was further illustrated^37,38^ that the dependency on imperfection distribution can be avoided if one uses an appropriately sized periodic unit-cell model with only one imperfection at the center as shown in Fig. 1. Therefore, there is a high degree of generality of the present numerical approach.

The simulations were performed under displacement control with a full range of biaxial compression considered. The boundary conditions and applied displacement directions are schematically shown in Fig. 1b, c. The roller boundary condition is imposed on faces *z* = 0 and *x* = 0 with only tangential slide allowed. The node at the origin (a corner point at the bottom of the substrate) is entirely fixed in space. The faces $z = W_{z}$ and $x = W_{x}$ are constrained to remain perpendicular to the *z* and *x* axes, respectively, during deformation^47^; and the bottom-substrate and top-film faces are traction-free. The compressive displacement was applied incrementally with its magnitude varying as $0 \le u_{x} \le \overline{u}_{x}$ (and similarly $0 \le u_{z} \le \overline{u}_{z}$), where $\overline{u}_{x}$ and $\overline{u}_{z}$ are the maximum applied displacements in *x* and *z* directions, respectively. The displacement increments for the static analysis were kept sufficiently small to avoid the potential increment-size dependency of the solutions^37^. The applied nominal strain therefore varies as $0 \le e_{xx} \le \overline{e}_{xx}$ (and $0 \le e_{zz} \le \overline{e}_{zz}$) where $\overline{e}_{xx}$ and $\overline{e}_{zz}$ are the maximum applied compressive strains in *x* and *z* directions, respectively. The biaxiality ratio (BR) is then defined as

7 $${\text{BR}} = \frac{{\overline{e}_{zz} }}{{\overline{e}_{xx} }} = \frac{{\overline{u}_{z} }}{{\overline{u}_{x} }}.$$

Due to the square geometry in the *xz*-plane, the strain ratio can also be written in terms of the applied displacements ($\overline{u}_{z}$ and $\overline{u}_{x}$) as seen in Eq. (7). Simulations for various biaxiality ratios were performed in that $\overline{u}_{x}$ was kept constant and $\overline{u}_{z}$ was altered within the range of $0 \le \overline{u}_{z} \le \overline{u}_{x}$ for different cases of BR. As a consequence, the biaxiality varies within the range of $0 \le {\text{BR}} \le$ 1, where ${\text{BR}} = 0$ pertains to uniaxial compression (applied in the *x*-direction) and ${\text{BR}} = 1$ corresponds to the perfectly equi-biaxial compression. Any other BR values represent non-equi-biaxial loading conditions.

The simulations were performed using the finite element software package ABAQUS (Version 2017, Dassault Systems Simulia Corp., Johnston, RI, USA). The 20-noded second-order continuum brick elements were used throughout the model. A uniform element distribution was used for the film layer (four layers of elements along the thickness of the film). A graded element distribution is applied for the substrate, with the element size increasing gradually from top (interface) to bottom. The detailed procedure of mesh generation and element definition can be found in the ABAQUS user’s manual^48^. To stabilize the numerical solutions in the post-buckling state, viscous damping was included and the corresponding damping factor was calculated via the adaptive automatic stabilization scheme^48^ for each time-increment via an iterative process until the converged solution is ensured. The iteration is controlled by the convergence history and the ratio of the energy dissipated by the viscous damping to the total strain energy (termed “accuracy tolerance”)^48^. In this study the accuracy tolerance was specified to be less than 10% to prevent damping-dependent solutions. Note that incorporating damping is discretionary, however it facilitates more efficient computations for large-scale simulations. It is worth mentioning that viscous damping was not employed in our earlier studies featuring problems with smaller scopes^35–38^.

The simulations were conducted using the Message Passing Interface (MPI) parallelization technique, and were implemented at the Center for Advanced Research Computing (CARC) at University of New Mexico. A total of 50 computing nodes (400 Intel-Xenon cores) were employed for each simulation. Preliminary analyses have verified that the numerical solutions were independent of the parallel computational procedures.

## Results and discussion

### Model verification

**Figure 2:**
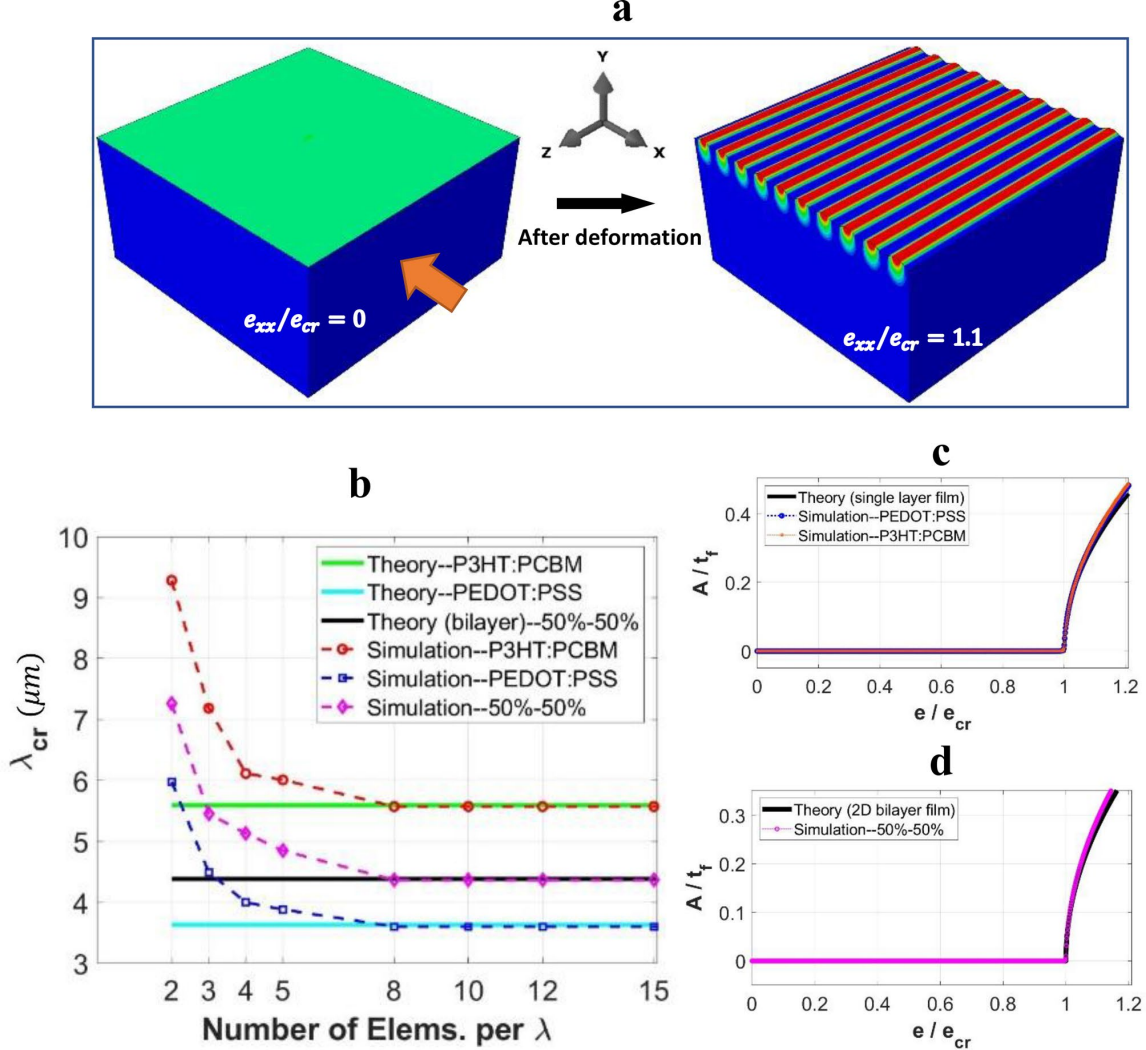
(**a**) Typical form of simulated 1D wrinkles associated with the converged mesh. (**b**) Variation of simulated 1D wrinkling wavelength with the number of elements per analytical wavelength. (**c, d**) Amplitudes of the sinusoidal wrinkles normalized by film thickness as a function of the applied strain normalized by the critical value, plotted for (**c**) single-layer, and (**d**) bi-layer film systems.

Verification of the numerical models in conjunction with the mesh convergence analysis are presented first. Consider uniaxial compression (BR = 0) applied to the film-substrate system. A typical form of simulated 1D wrinkles using the converged mesh is shown in Fig. 2a. The mesh refinement scheme is based on the number of elements in the *x* and *z* directions per critical wavelength of the 1D mode ($\lambda_{cr}$ in Eq. 1), and equal mesh size in both *x* and *z* is maintained to ensure a square element shape in the plane. This refinement practice provides a neat control over the total number of elements in a wrinkled structure. Figure 2b shows the simulated critical wavelength of the 1D mode as a function of the number of elements per unit wavelength. The theoretical values are also included as horizontal lines for comparison. Three different thin-film materials were studied: a single-layer P3HT:PCBM, a single-layer PEDOT:PSS, and a composite (bi-layer) film comprising of 50%–50% (equal thickness) P3HT:PCBM on top of PEDOT:PSS. The case of composite film has been comprehensively studied in our earlier 2D planar models^36,37^, and is now successfully extended to the full 3D model. (Note that the theoretical relations, Eqs. (1–3) and (6), valid for a single-layer film, need to be revised for a bi-layer film via the definition of effective composite film moduli under bending and axial deformations^2,49,50^. For brevity they are not listed here.) As can be seen from Fig. 2b, mesh-insensitive solutions were achieved when the mesh density reaches about eight elements per unit wave. Further increases in mesh density result in constant critical wavelengths matching the theoretical values.

Figure 2c shows how the amplitude of the 1D wrinkles (normalized by the film thickness) evolves with the applied strain (normalized by the critical value at the onset of primary bifurcation), for the two cases of single-layer film material. The mesh density used is the converged 10 elements per wavelength. The corresponding plot for the bi-layer composite film is shown in Fig. 2d. The theoretical response displayed in Fig. 2c, d are based on Eq. (6). It is evident that the numerical results are generally in agreement with the analytical solutions in all cases. From the discussion in Supplementary Information, the critical wavelength of 1D wrinkles is the shortest wavelength compared to all other primary and post-buckling modes under general biaxial loading. In the remainder of this paper, all simulation results were based on the sufficiently fine mesh of 10 elements per wavelength to ensure accuracy.

**Figure 3:**
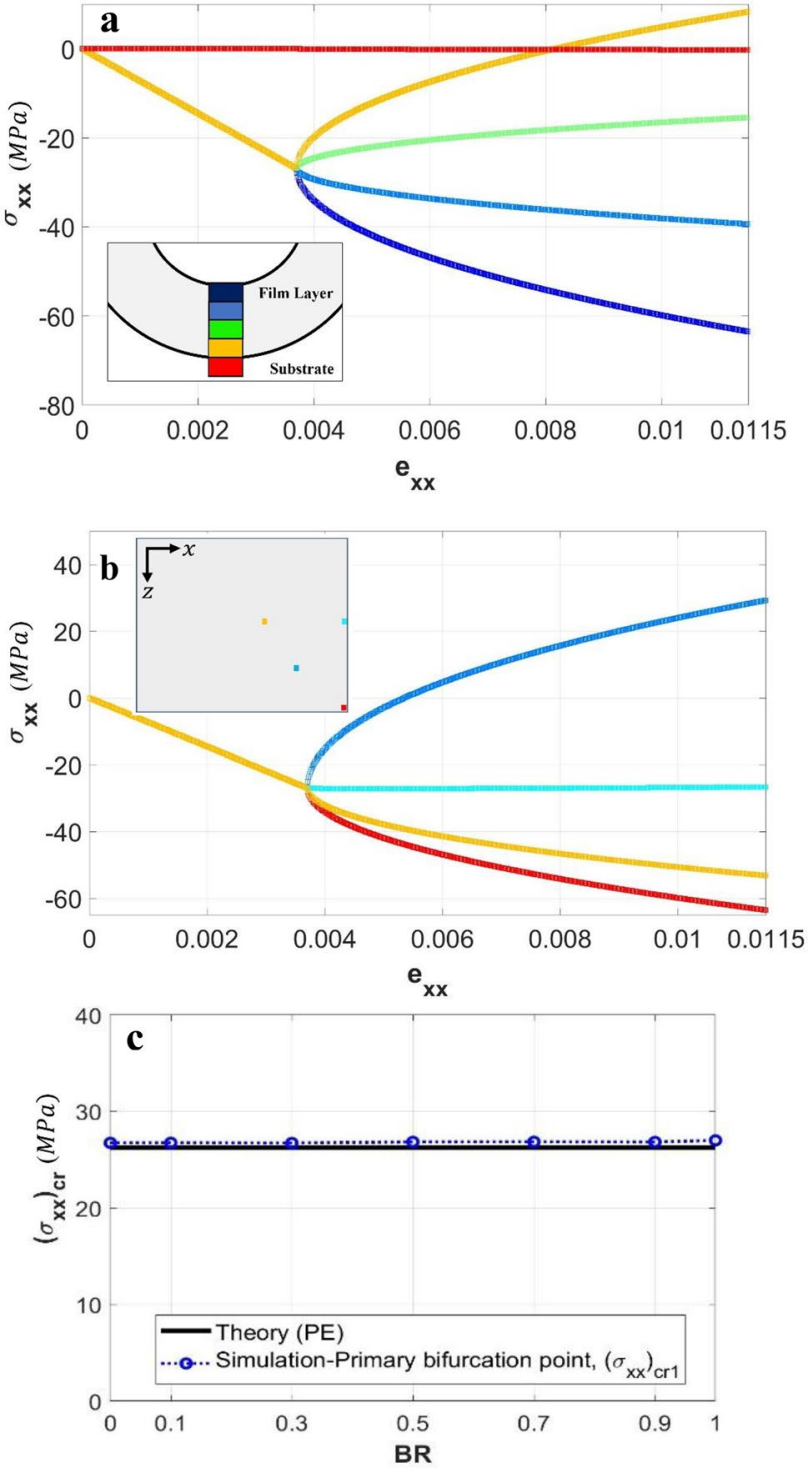
(**a, b**) Evolution of stress ${\sigma }_{xx}$ as a function of applied strain ${e}_{xx}$, (**a**) through the film thickness and into the top element of the substrate at a chosen location, and (**b**) at various locations of the film’s top surface. (**c**) Variation of critical stress, ${({\sigma }_{xx})}_{cr}$, with the biaxiality ratio, BR. The theoretical solution is also included for comparison.

As a part of the verification study, we now present the evolution of stresses using the case of P3HT:PCBM film under uniaxial loading (BR = 0). A column of elements along the film-thickness direction from top (free surface) to bottom of film (adjacent to interface) and into the top substrate element, near the lower-right corner of Fig. 1c, were selected. Figure 3a shows the history of the elemental stress component $\sigma_{xx}$ in all these elements (evaluated at the elements’ centroids) as a function of the applied strain in the *x*-direction, $e_{xx}$. It is clear that the stress developments in all the film elements considered are identical at the pre-instability stage, and bifurcation starts at the same point with the $\sigma_{xx}$ value in agreement with Eq. (2). Upon instability the top and bottom film elements selected are at the concave and convex sides of a wrinkle, respectively, so their stresses become more and less negative, respectively, as deformation progresses. Note that the stress in the compliant substrate immediately below the interface remains close to zero throughout the deformation. The same type of stress history is plotted in Fig. 3b, using four random elements at the top surface of the film. The locations of the chosen elements are highlighted in the inset of Fig. 3b. Again, bifurcation initiates at the same point consistent with the analytical critical stress value. The post-instability stress diverges depending on the element location.

In addition to uniaxial loading, the critical stresses associated with the primary bifurcation mode for various biaxiality ratios were obtained from the numerical simulations and compared with the theoretical value of Eq. (2). The variation of the magnitude of $\left( {\sigma_{xx} } \right)_{cr}$ with the BR value is shown in Fig. 3c. As can be seen, the simulated $\left( {\sigma_{xx} } \right)_{cr}$ is independent of the biaxiality ratio and is close to the theoretical prediction. It is worth mentioning that, for biaxial loading, $\left( {\sigma_{zz} } \right)_{cr}$ will change with BR so overall the critical stress state is actually a function of the load biaxiality.

### Wrinkle patterns vs. macroscopic response

**Figure 4:**
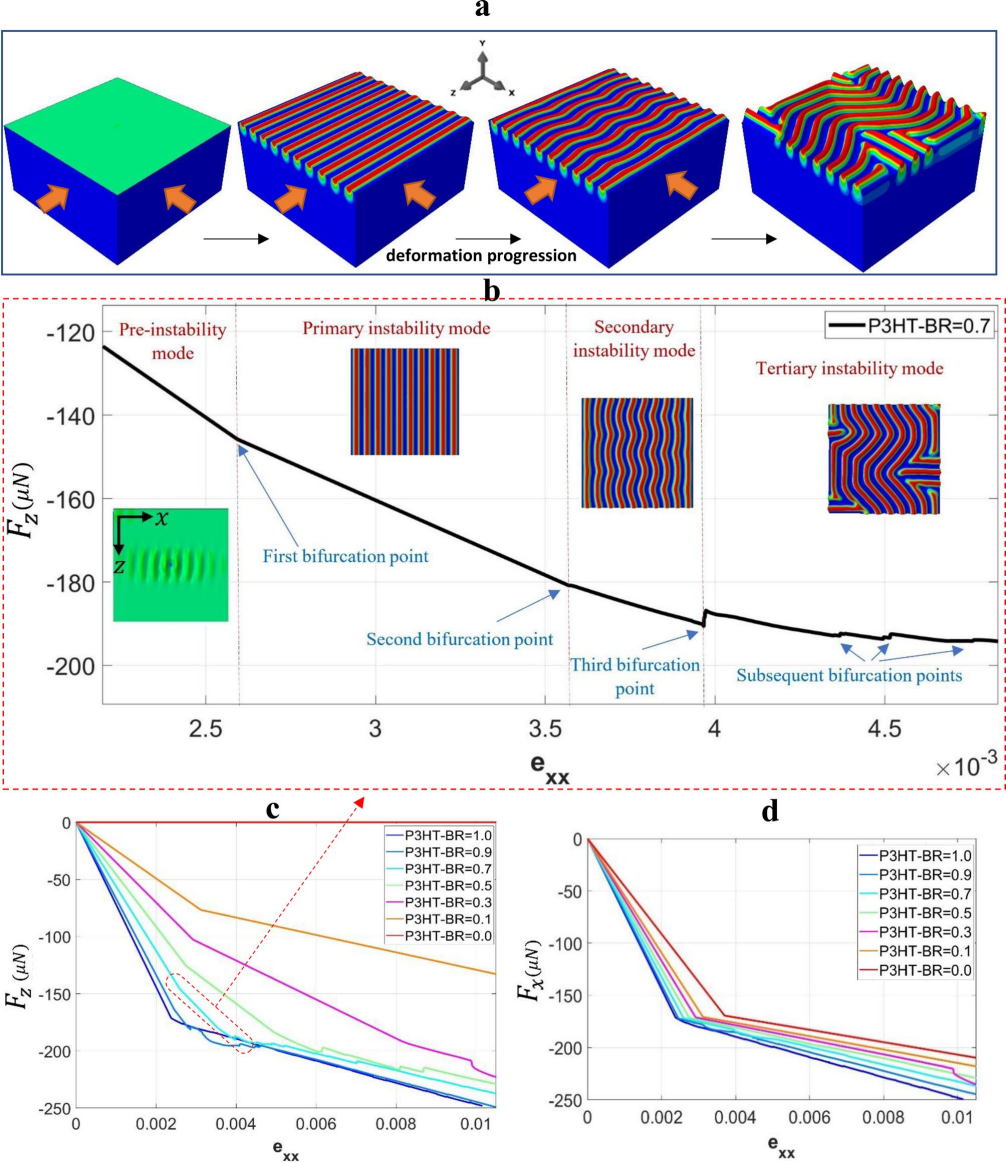
(**a**, **b**) Evolution of surface wrinkling patterns during the loading history, starting from a flat surface to 1D mode (primary), herringbone (secondary), and then to labyrinth (tertiary) mode, obtained from a single simulation run for the case of non-equi-biaxial loading with BR = 0.7. (**c**, **d**) Variation of reaction force (**c**) ${F}_{z}$ and (**d**) ${F}_{x}$ with the applied strain (${e}_{xx}$); the results for various BR values are included for comparison.

The present work investigates the full range of biaxial loading between BR = 0 and 1. In this section we first present the simulated surface morphologies and their correlation with the macroscopic load response, using a special case of non-equi-biaxial loading with BR = 0.7. While the case of BR = 0.7 was chosen arbitrarily, this loading ratio does lead to a series of pattern changes from 1D mode (primary) to herringbone (secondary) and then to labyrinth (tertiary), as shown in Fig. 4a. The entire sequence from the pre-instability stage can be directly captured in one simulation run. From the model output, the reaction forces in either compressive direction may be plotted against the applied strain. An example is shown in Fig. 4b using the reaction force in *z*, *F*_*z*_, and the applied strain *e*_*xx*_. The curve spans a wide range of deformation history from pre-instability to well into the tertiary instability mode, with the representative top-view surface patterns included as insets in the figure. Whenever the instability mode change occurs, the curve displays a sudden change in slope or load drop.

Before the onset of instability, the film-substrate system is under nominally uniform compression and the surface remains generally flat. In Fig. 4b, we include a pre-instability image with a highly amplified displacement map, illustrating that the formation of wrinkles is in fact a gradual process starting from very small disturbances around the imperfection site^38^. The onset of primary instability is at the applied strain of approximately 2.6 × 10^–3^, and the dominant 1D wrinkle mode lasts through the strain of approximately 3.6 × 10^–3^ when the secondary bifurcation (herringbone mode) commences. As the strain approaches 4.0 × 10^–3^, an abrupt load reduction occurs which signifies a significant change in pattern into the labyrinth mode. Subsequent aberrations of the curve correspond to further adjustments of wrinkle configurations which will change the labyrinth form as the applied strain continues to increase. The simulation approach adopted here enables continuous visualization of the evolving instability patterns and the correlation with the macroscopic mechanical response.

It should be noted that the bifurcation points identified in Fig. 4b depend on the material properties and loading condition. The specific case of P3HT:PCBM film with BR = 0.7 is chosen for illustration. Figure 4c shows the reaction force *F*_*z*_ versus applied strain *e*_*xx*_ for seven biaxiality ratios ranging from 0 to 1. The corresponding plot for *F*_*x*_ is shown in Fig. 4d. The *e*_*xx*_ value is used as a reference for both figures to represent the extent of applied strain. In Fig. 4c the case of BR = 0 is for uniaxial loading along the *x*-direction so *F*_*z*_ remains zero throughout the history. From Fig. 4c, d, a linear elastic response is observed at the pre-instability stage in all cases. In Fig. 4d a higher BR value results in a higher initial slope due to the Poisson’s ratio effect of biaxial loading. For each BR the first change in the load-strain slope corresponds to the onset of the primary instability mode. Subsequent irregularities along the curves are the result of mode transformation or configuration change, as described in the previous paragraph. (Note that the curve in Fig. 4b is a zoomed-in view of the curve of BR = 0.7 in Fig. 4c.) The curves in Fig. 4c, d indicate that different biaxiality ratios result in significantly different post-instability behavior. The following section is then devoted to a systematic analysis of the wrinkle pattern formation over the entire BR span.

### Wrinkle patterns versus biaxiality ratio

**Figure 5:**
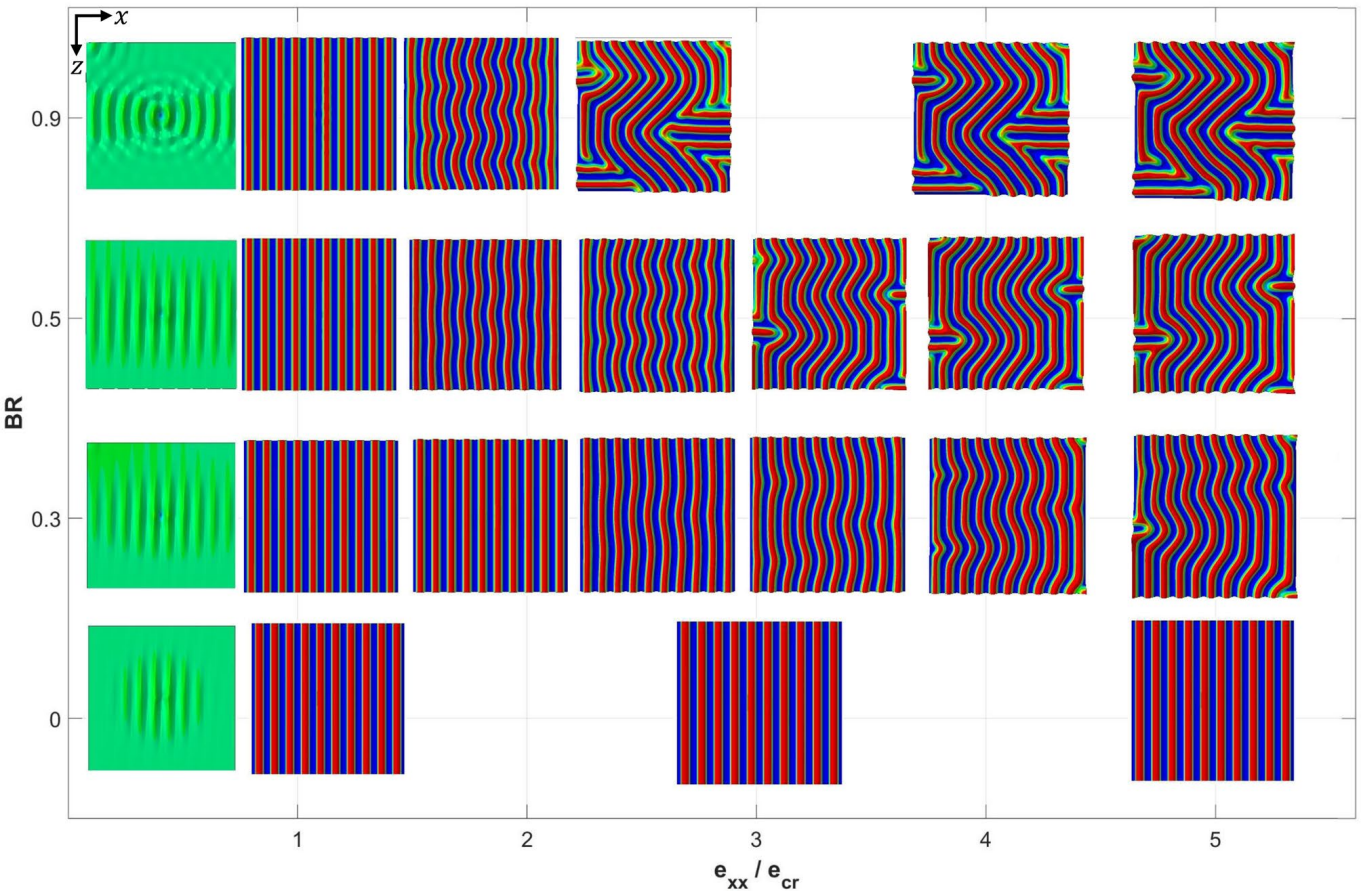
Evolution of wrinkling patterns as a function of applied strain (normalized by the critical value) and biaxiality ratios within the range of $0\le BR\le 0.9$.

To assess the dependency of wrinkle patterns on the biaxiality ratio, extensive simulations were conducted with the pattern evolution closely monitored. Due to the nature of the resulting patterns as discussed below, the cases of 0.9 < BR $\le$ 1.0 require more detailed examinations. Therefore, this range is to be presented separately below. The progression of deformation is characterized by the normalized applied strain in the *x*-direction, $e_{xx} /e_{cr}$, where $e_{cr}$ is the numerically obtained critical strain corresponding to the first bifurcation point. Figure 5 shows a collection of top-view (*xz*-plane) wrinkle patterns displayed by the P3HT:PCBM film, for biaxiality ratios between 0 and 0.9 up through an applied strain of $e_{xx} /e_{cr} = 5$. The color contours represent the out-of-plane displacement ($u_{y}$) with the red and blue colors being the highest (peak) and lowest (valley) positions, respectively. Note that the same quantitative color scheme is applied to all the surface patterns presented in this paper.

In Fig. 5 the left-most images are patterns right before instability, at $e_{xx} /e_{cr} \approx 0.98$, showing very small disturbances originating from the imperfection site. (A very high scaling factor of 1000 is assigned to these pre-instability images to make them visible.) Shortly after the deformation reaches $e_{xx} /e_{cr} = 1.0$, all cases of BR from 0 to 0.9 have displayed well defined 1D wrinkles. For high BR values such as 0.9, the wrinkles are perpendicular to the *x*-direction since *e*_*xx*_ was set to be the higher compressive strain than *e*_*zz*_ in the models. For uniaxial loading (BR = 0), the 1D mode persists throughout the entire history. For biaxial loading with a non-zero *e*_*zz*_ component, the parallel wrinkles will start to curve so as to transform into the herringbone-like structure as deformation continues. This transformation occurs earlier as the biaxial loading becomes more prominent (increasing BR). In addition, another major post-buckling transformation from herringbone to labyrinth appears for high BR values, which also tends to occur earlier as BR increases.

The herringbone pattern observed in Fig. 5 deserves further discussion. As stated in Supplementary Information, theoretical uncertainties exist in the literature regarding the evolution of herringbone pattern and the necessary loading condition in the post-instability regime. From the current simulations, the pattern is essentially an outcome of lateral undulation (post-bifurcation) in the 1D waves. In addition, this mode was not observed under pure equi-biaxial condition and was captured only for non-equi-biaxial cases of $0 < {\text{BR}} \le { }$ 0.90. It was observed that the short wavelength (perpendicular to the local wrinkle lines) remains invariant, but the long wavelength (lateral undulation) and the jog angle depend strongly on the applied strain. Our results about the herringbone pattern in general conform to the theoretical considerations in references^19,21,23^.

**Figure 6:**
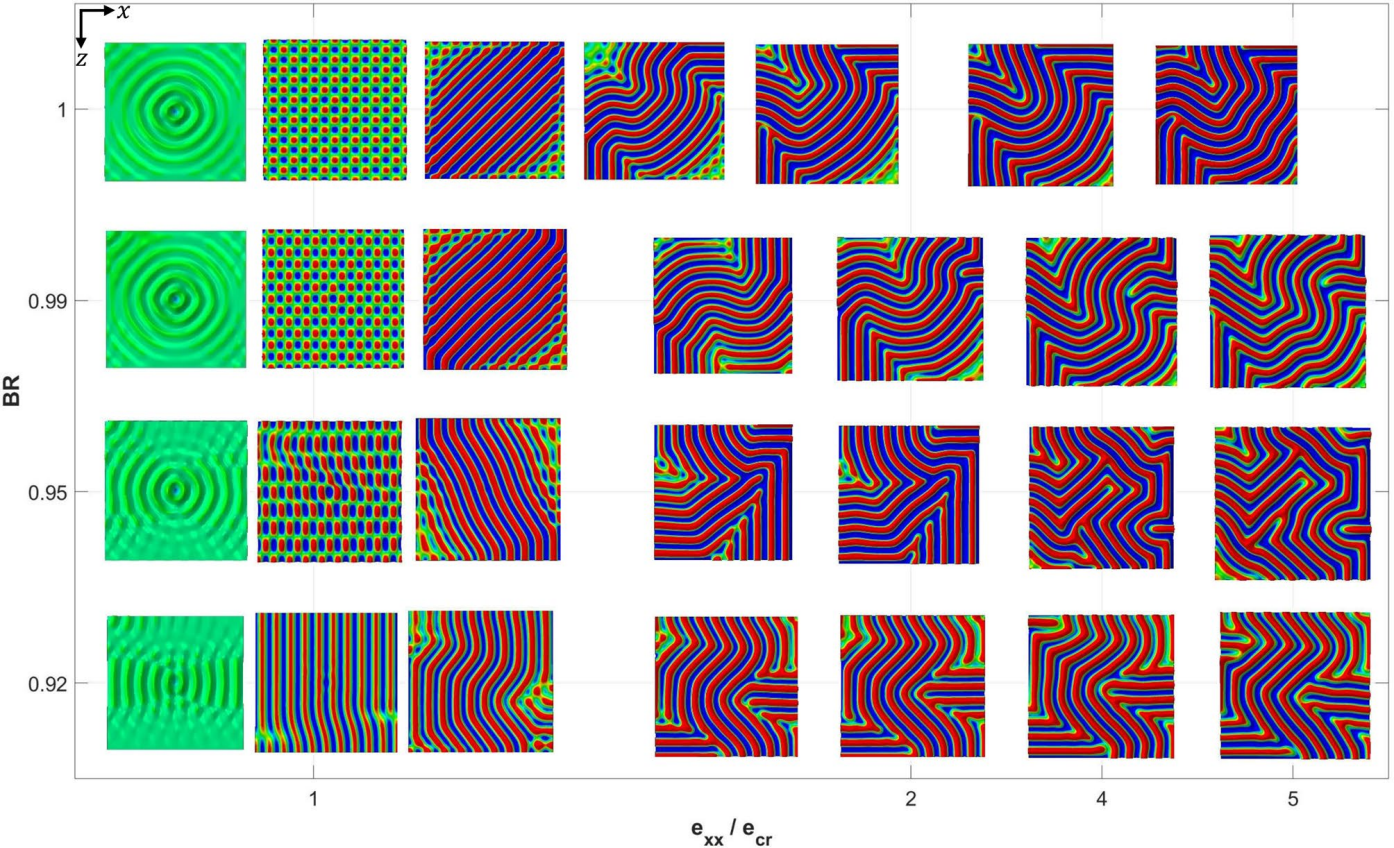
Evolution of wrinkling patterns as a function of applied strain (normalized by the critical value) and biaxiality ratios within the range of $0.9<BR\le 1.0$.

Figure 6 shows the wrinkle patterns at various stages of deformation, for BR values greater than 0.9. With the loading conditions now closer to equi-biaxial, it can be seen that the 1D mode observed previously in Fig. 5 is suppressed right from the onset of first bifurcation. (Immediately before instability a very small disturbance in the form of ripples originating from the imperfection site can be detected under a very high scaling factor.) There is a tendency for the primary instability mode to become checkerboard-like as BR approaches unity. Soon after the primary mode, there is a transient stage where the checkerboard form breaks down and more continuous wrinkles emerge leading to a labyrinth structure. This transformation happens within the strain range of $1 \le e_{xx} /e_{cr} \le 2$, with the labyrinth mode staying dominant thereafter. Note that the transient patterns seen in Fig. 6 may be interpreted as a hybrid mode of checkerboard-herringbone; the term “bistable modes” have been used in the literature to refer to such hybrid instability patterns^21,51^.

**Figure 7:**
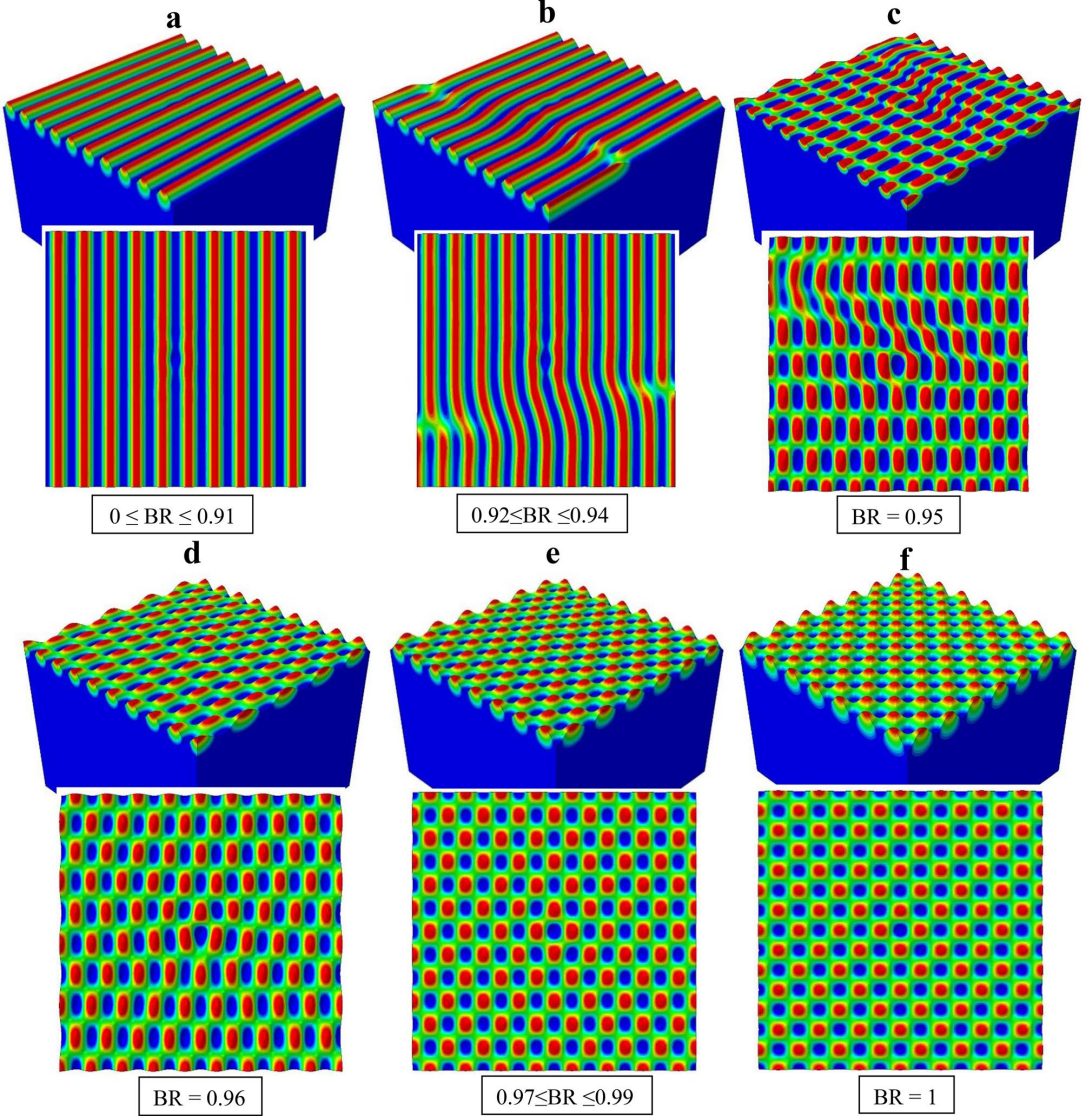
Primary deformation instability patterns obtained for various biaxiality ratios.

**Figure 8:**
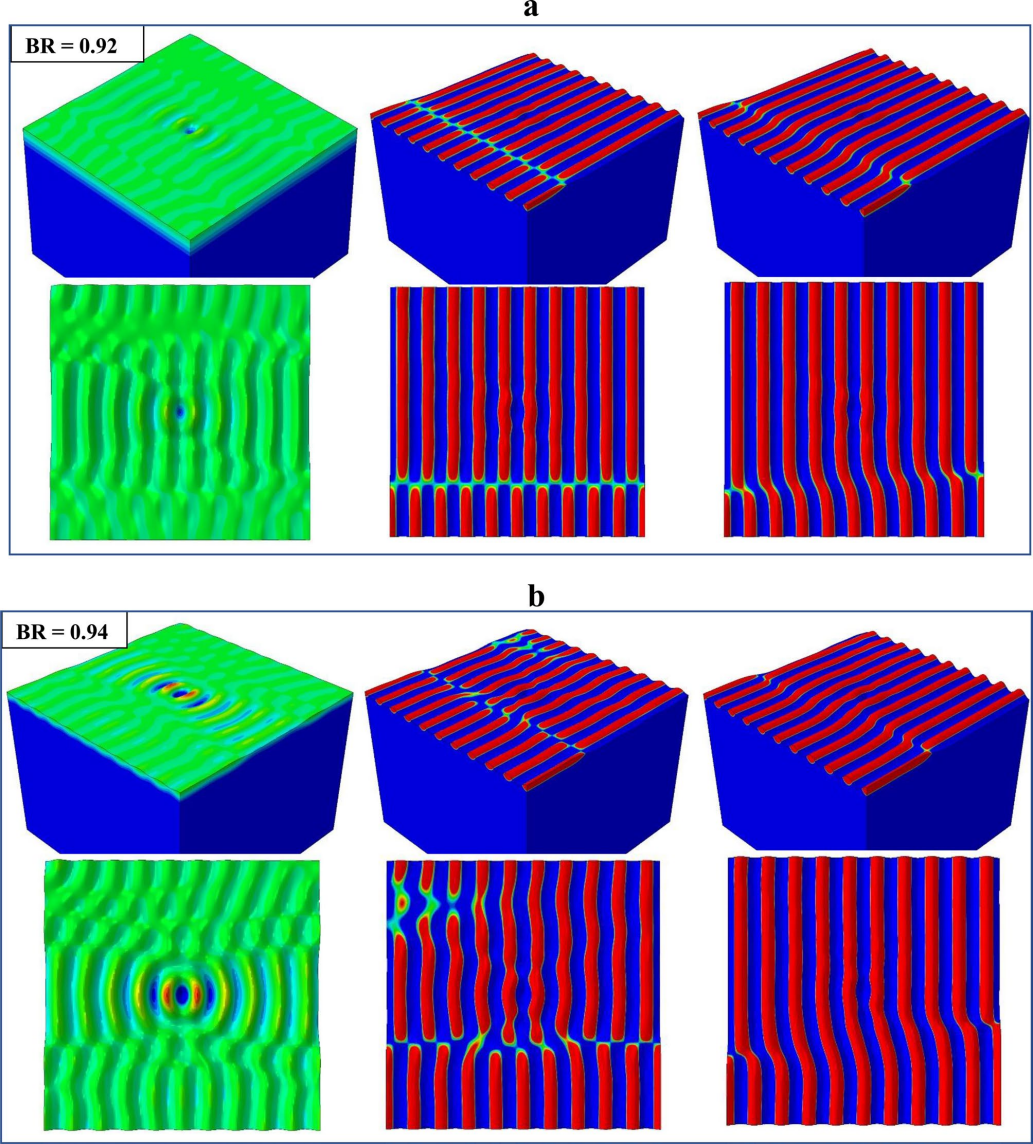
Formation and progression of “branched” wrinkles through the link-up process under the influence of loading biaxiality. (**a**) BR = 0.92 and (**b**) BR = 0.94.

The primary instability modes observed in Figs. 5 and 6 are now grouped together in Fig. 7 for a clear comparison. Within the range of $0 \le {\text{BR}} \le$ 0.91 (Fig. 7a) the mode is 1D wrinkles. It is worthy of note that, for a biaxial loading state with the applied compressive strain in one direction (*z* in the current study) as high as above 90% of that in the other direction (*x*), the primary instability mode is still 1D wrinkles. Some 1D wrinkles become “branched” when BR is increased to the range of $0.92 \le {\text{BR}} \le$ 0.94, which is also associated with the lateral kinks displayed by most of the waves (Fig. 7b). This is a transitional state from the 1D wrinkle to checkerboard modes. Figure 8a, b shows the evolution of branching patterns for BR = 0.92 and 0.94, respectively. In both cases the 1D wrinkles at the two opposite sides of the model appear to emerge in a staggered manner. With further deformation the two sets of waves link up, resulting in a kinked and branched structure. As BR is increased to 0.95 (Fig. 7c), the continuous waves are entirely broken up by the increasingly dominant kinking and branching, and the surface topography tends toward discrete islands.

Beyond BR = 0.95, a periodic wave form in both the *x*- and *z*-directions becomes more distinct and the checkerboard pattern takes shape. Note that the true square-checkerboard is obtained only when BR = 1 (Fig. 7f). With a slight deviation from perfect equi-biaxiality, e.g., at BR ≈ 0.99, the wavelength along the *z*-direction is greater than that along the *x*-direction (Fig. 7e). It is worth mentioning that this non-square checkerboard pattern is qualitatively akin to the hexagonal/triangular modes proposed analytically by Cai et al^17^. Major findings from Fig. 7 can be summarized as follows:

- The wrinkling patterns are highly sensitive to the loading biaxiality, and variations of the checkerboard pattern are observed for BR $\ge$ 0.95. All these simulation results are with an initially perfectly flat surface, influenced only by the load biaxiality. This aspect has not been predicted by any theoretical studies discussed in Supplementary Information.
- With BR $\ge$ 0.95 the loading state is very close to the pure equi-biaxial compression. This may explain why the perfect square-checkerboard pattern has not been observed in earlier experimental studies^1,3,17^ (in addition to other possible reasons discussed in the literature), since a nominal equi-biaxial state in actual experiments is likely to have slight deviations so non-square types of checkerboard patterns may be easily triggered. Furthermore, any non-uniformity in the film and substrate materials may also affect the local stress state.

### Construction of instability phase diagrams

**Figure 9:**
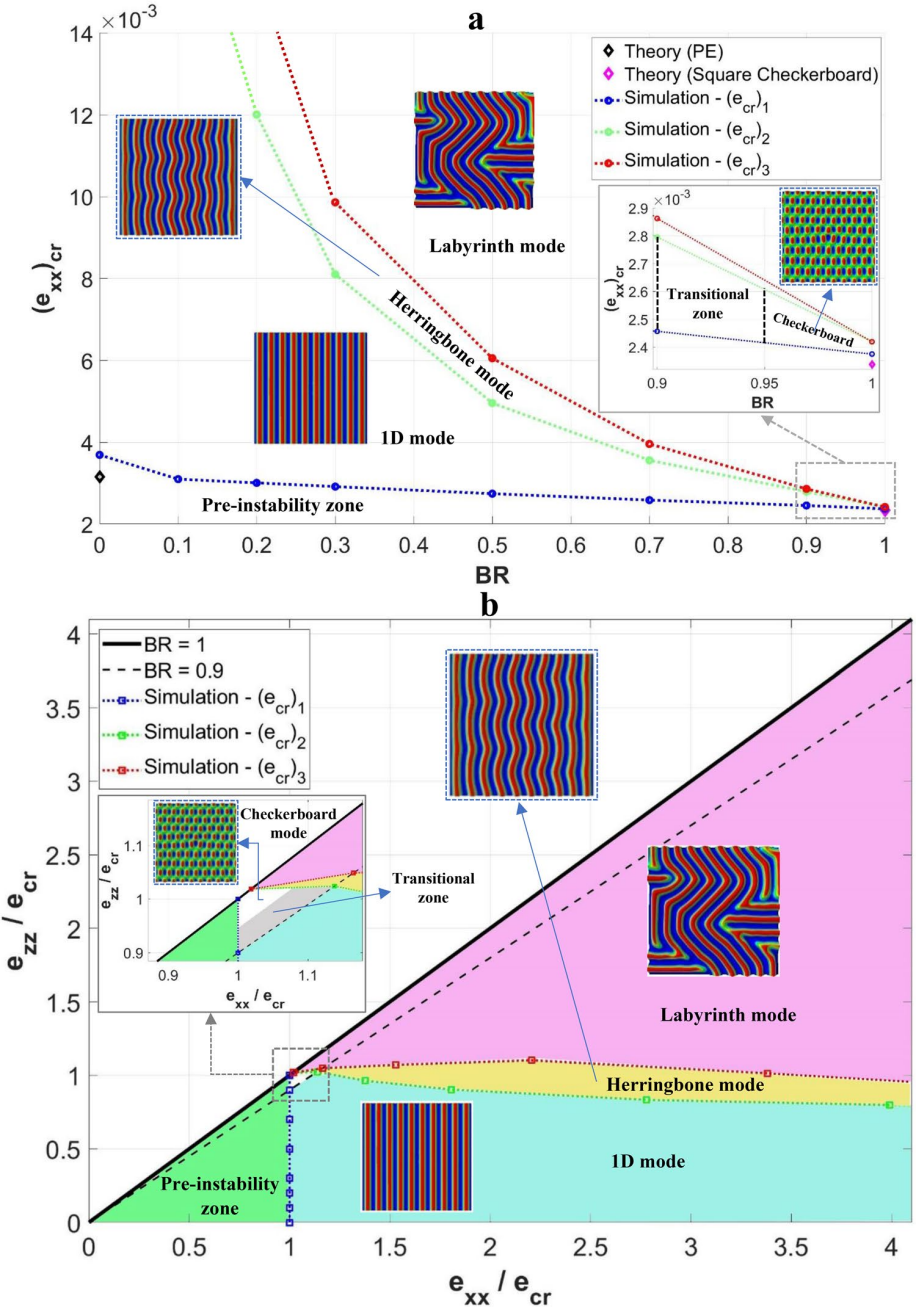
(**a**) Variation of numerically calculated critical strain, ${({e}_{xx})}_{cr}$, with the biaxiality ratio, BR; critical strains corresponding to the first three bifurcation points are shown and respectively labeled as ${({e}_{cr})}_{1}$, ${({e}_{cr})}_{2}$, and ${({e}_{cr})}_{3}$. (**b**) Instability phase diagrams.

The results presented in Figs. 5 and 6 provided an overview of how the instability pattern evolves as deformation progresses for the various biaxiality states. Since our simulations are able to predict temporal evolution of one bifurcation mode to another, the critical biaxial strains for each mode may be graphically presented so as to create a phase diagram. Figure 9a shows the variation of critical strains in the *x*-direction, $\left( {e_{xx} { }} \right)_{cr}$, as a function of biaxiality ratio for our model system of P3HT:PCBM film on PDMS substrate. (Different materials will lead to different quantitative values but the qualitative features remain the same, as confirmed by our analyses using the PEDOT:PSS thin film.) Note that, with any combination of BR and $\left( {e_{xx} { }} \right)_{cr}$ values in Fig. 9a, $\left( {e_{zz} { }} \right)_{cr}$ is also determined. The critical strains here were obtained from the load-strain curves in conjunction with the wrinkling patterns, as discussed in previous sections. The critical values for the primary, secondary, and tertiary bifurcations are denoted as $\left( {e_{cr} } \right)_{1}$, $\left( {e_{cr} } \right)_{2}$, and $\left( {e_{cr} } \right)_{3}$, respectively. These curves serve as the boundaries between the domains whose characteristic instability patterns are also displayed. Two theoretical values for the first bifurcation are included for comparison, one for the square-checkerboard mode and the other for 1D wrinkles under the plane strain condition.

It is evident from Fig. 9a that the critical strains decrease as the biaxiality ratio increases. These domain boundaries converge toward the equi-biaxial state (BR = 1). However, at BR = 1 the three critical values do not actually coincide, as shown in the inset. There is a very small primary instability region where the characteristic pattern is checkerboard. The critical strains, $\left( {e_{cr} } \right)_{2}$ and $\left( {e_{cr} } \right)_{3}$, on the other hand, are indistinguishable and thus the square-checkerboard transforms to labyrinth as the compressive strain increases. In the vast majority of biaxial loading conditions, 1D wrinkling is the primary bifurcation mode. Herringbone exists between 1D wrinkles and labyrinth. The stable herringbone pattern, however, occurs only within a relatively narrow region in this diagram.

An alternative form of the phase diagram is shown in Fig. 9b, using $e_{xx}$ and $e_{zz}$ to characterize the biaxial state (note that both axes are normalized by $\left( {e_{cr} } \right)_{1}$ along the *x*-direction). The diagonal line represents the border of this diagram, since $e_{xx} \ge e_{zz}$ in all cases of this study without the loss of generality. (Flipping the loading directions will lead to exactly the same results but in the other half of the figure). A dashed line representing BR = 0.9 is added in the figure as a reference (not as a “phase boundary”), to aid in the reading of the map. The horizontal axis itself corresponds to the case of BR = 0. The phase boundaries are the three critical strains $\left( {e_{cr} } \right)_{1}$, $\left( {e_{cr} } \right)_{2}$ and $\left( {e_{cr} } \right)_{3}$ normalized by $\left( {e_{cr} } \right)_{1}$ (corresponding to the respective BR values). As in Fig. 9a, the three main domains featured in Fig. 9b have the characteristic instability patterns of 1D wrinkles, herringbone and labyrinth. The checkerboard pattern, as well as the transitional hybrid mode of checkerboard-herringbone mentioned in the last section, only exist in a very small region when BR > 0.95 between the pre-instability and labyrinth domains. For all other BR values, the development of bifurcation modes follows the order of 1D wrinkles, herringbone and labyrinth as deformation progresses.

## Concluding remarks

The embedded imperfection approach is successfully applied to three dimensions, and a comprehensive study on the surface instability patterns induced by biaxial compression is presented. The technique is easily implemented for structures consisting of thin films above a compliant substrate. Instead of treating the surface patterns discretely under specific assumptions, the generation of wrinkling morphologies and their transformations can be directly obtained from the simulations. The results provide insight and mechanistic rationale for uncertainties seen from past theoretical and experimental considerations. The state of biaxiality is found to influence the surface pattern significantly, and each bifurcation mode can be traced back to certain abrupt changes in the overall load–displacement response. The square-checkerboard pattern has proven to be a dominant bifurcation mode only under strict equi-biaxial loading within a very narrow range of strains; physical experiments would easily miss the condition due to any slight deviation. A lower biaxiality ratio (closer to uniaxial loading) expands the dominance of the 1D wrinkles. However, for a biaxiality ratio as high as 0.9, the primary bifurcation mode is still 1D wrinkles except that it transforms into herringbone and then labyrinth patterns quickly. The phase diagrams constructed from the simulation results provide an overview of wrinkling configurations over the entire span of biaxial loading. It is worth emphasizing that all the results presented in the current paper are based on simple isotropic linear elastic material behavior with an initially perfectly flat film layer. The 3D numerical model is well suited for future investigations involving more complex material, geometric and loading conditions.

## Supplementary Information

Supplementary Information.

## Data Availability

All data generated and analyzed during the current study are included in this article.
