# Supplementary material for: Direct numerical simulations of three-dimensional surface instability patterns in thin film-compliant substrate structures
Source: Sci Rep. 2021 Aug 12;11:16449. doi: 10.1038/s41598-021-95414-8 (PMC8361117; doi:10.1038/s41598-021-95414-8)
Supplement: Supplementary file 1 — Supplementary Information. [file 41598_2021_95414_MOESM1_ESM.docx]

**Supplementary Information**

Various theoretical formulations of surface instability are available in the literature. Historically, studies by Allen^1^ and Koiter^2^ on the buckling of structural sandwich panels, and Biot^3, 4^ on the deformation instabilities of elastic half-spaces, served as the foundation of more recent work. The main goal of the analytical approaches is typically to characterize the wrinkling parameters including wavelength, amplitude and critical stress/strain etc. For surface wrinkling of a thin film on top of a compliant substrate, one may categorize available analytical techniques into generic groups of 1D^3, 5, 6, 7, 8^ and 2D buckling formulations^9, 10, 11, 12, 13, 14, 15, 16^. The 1D solutions, based on the plane strain assumption, focus on the classical sinusoidal wrinkles (also termed 1D wrinkles or cylindrical wrinkles in the literature). The 2D analytical solutions allow for in-plane biaxial loading, and they were mostly developed on the foundation of nonlinear Föppl–von Kármán partial differential equations (also known as von Karman plate theory), with the film layer being considered as a nonlinear thin flat plate. Simplification was made in the strain field (namely von Kármán strain tensor) in that, compared to the Green strain tensor in full 3D continuum, all the nonlinear and higher-order terms associated with the in-plane displacement components were neglected^17, 18^. This assumption requires the thin film thickness to stay constant. In the context of surface wrinkling of the film-substrate structure, the prediction may be valid only for small strains and the cases where the film thickness is very small compared to the nominal wrinkling wavelength and the substrate thickness. Here we discuss representative theoretical solutions for surface wrinkling, some of which are used for numerical model verifications in the current study.

**One Dimensional Wrinkles**

Consider the thin film-substrate structure shown in Fig. 1 of the main paper. Under uniaxial compression, 1D sinusoidal wrinkling commences once the critical point for instability is reached. Assuming that the substrate is semi-infinite, film and substrate are fully bonded at the interface, and both layers are linear-elastic and isotropic, the wavelength of the wrinkles at the onset of bifurcation (primary instability mode) follows

$$\left( \lambda_{cr} \right)_{1D}=\lambda_{cr}=2\pi t_{f}\left[ \frac{E_{f}}{3\left( 1-\upsilon_{f}^{2} \right)\bar{E}_{s}} \right]^{1/3} , (1)$$

where $\lambda_{cr}$ is the critical wavelength of the 1D mode, and $t_{f},$ $E_{f}$, and $\nu_{f}$ are, respectively, thickness, Young’s modulus, and Poisson’s ratio of the film layer. $\bar{E}_{s}$ is an elastic parameter of the substrate and will be defined later in this section. In terms of wave number,$k={2\pi}/\lambda$, one may rewrite Eq. (1) as

$$k_{cr}=\left( \frac{1}{t_{f}} \right)\left[ \frac{3\left( 1-\upsilon_{f}^{2} \right)\bar{E}_{s}}{E_{f}} \right]^{1/3} . (2)$$

The well-known one-dimensional derivation of the critical wavelength of the sinusoidal wrinkles, Eq. (1), is based on the Euler-Bernoulli beam equations (modified by Biot constants^3^ to deal with the substrate effect), and was derived for uniaxial compression under the plane strain condition via the force balance approach^5, 6^. Alternatively, Eq. (1) can be derived from the linearized two-dimensional von Karman equations under uniaxial loading and by using the total energy minimization approach^9, 10, 12^. Note that there is an analogy between the modified Euler-Bernoulli beam equations and linearized von Karman plate equations, which leads to identical solutions for sinusoidal wrinkles (under the assumptions mentioned). The critical wrinkling stress$, \sigma_{cr}$, corresponding to $\lambda_{cr}$ was also derived as^1, 6, 10^

$$\sigma_{cr}=\left[ \frac{E_{f}}{4\left( 1-\upsilon_{f}^{2} \right)} \right]\left[ \frac{3\left( 1-\upsilon_{f}^{2} \right)\bar{E}_{s}}{E_{f}} \right]^{2/3}, (3)$$

assuming that the stress state is uniform in the film layer (with the cross-section area of $t_{f}\cdot w_{z}$ as shown in Fig. 1). It should be mentioned that Eqs. (1) to (3) are also the exact solutions of the “nonlinear” form of von Karman equations for a thin film on an elastic foundation^10^. The critical strain for 1D wrinkling, $e_{cr}$, was reported as^6, 10, 15^,

$$\left( e_{cr} \right)_{1D}=e_{cr}=\left( \frac{1}{4} \right)\left[ \frac{3\left( 1-\upsilon_{f}^{2} \right)\bar{E}_{s}}{E_{f}} \right]^{2/3}. (4)$$

Note that it is equivalent to the critical stress in Eq. (3) divided by the plane-strain modulus of the film, ${E_{f}}/\left( 1-\upsilon_{f}^{2} \right)$. Equation (4) cannot accurately capture the actual critical strain if the deformation deviates from the plane strain condition^19, 20^.

The substrate parameter, $\bar{E}_{s}$ in Eqs. (1) to (4), has different definitions in the literature depending on the assumptions made for the substrate itself and the interaction between the film and substrate. More commonly, the substrate parameter is defined based on the assumption of zero tangential traction components at the film-substrate interface, leading to $\bar{E}_{s}$= ${E_{s}}/\left( 1-\upsilon_{s}^{2} \right)$, where $E_{s}$ and $\nu_{s}$ are, respectively, Young’s modulus and Poisson’s ratio of the substrate material. On the other hand, under the assumption of zero in-plane displacement components at the film-substrate interface (instead of traction), $\bar{E}_{s}$ is derived^9, 11, 15^ as $\bar{E}_{s}$= $\left[ {E_{s}}/\left( 1-\upsilon_{s}^{2} \right) \right]\cdot\left[ {4{(1-\upsilon_{s})}^{2}}/\left( 3-4\upsilon_{s} \right) \right]$. Note that the difference between the two forms of $\bar{E}_{s}$ is negligible if the substrate is near-incompressible; when it is fully incompressible ($\upsilon_{s}$= 0.5), ${4{(1-\upsilon_{s})}^{2}}/\left( 3-4\upsilon_{s} \right)=1$. Note that a different form of Eqs. (1) to (4) taking into account both the shear traction and tangential displacements at the film-substrate interface is also available in the literature^7^ (derived under the plane strain assumption with continuous tractions and displacements across the interface). However, its differences with Eqs. (1) to (4) are negligible when the substrate is near-incompressible ($\upsilon_{s}\cong$ 0.5). Also note that Eqs. (1) to (4) apply to the case of an infinitely thick substrate; analytical solutions for the case of finite substrate thickness are available^15, 21, 22^.

**Extension to Biaxial Compression**

We now consider the same film-substrate structure subjected to pure equi-biaxial compression. It has been shown that, under equi-biaxial compression, the square-checkerboard pattern has the lowest energy in the buckled state^9, 11^. The wavelength of square-checkerboard mode was derived^9, 10, 12^ as

$$\left( \lambda_{cr} \right)_{Cb}=\sqrt{2}\left( \lambda_{cr} \right)_{1D}=2\sqrt{2}\pi t_{f}\left[ \frac{E_{f}}{3\left( 1-\upsilon_{f}^{2} \right)\bar{E}_{s}} \right]^{1/3} . (5)$$

It was also postulated that the critical stress introduced in Eq. (3) not only applies to 1D wrinkles but also applies to any possible biaxial wrinkling mode that satisfies the relation of $\sqrt{k_{x}^{2}+k_{z}^{2}}=k_{cr}$ (any wrinkling shape that the general solution can be written as a linear combination of 1D modes), where $k_{cr}$ is defined in Eq. (2) ^9, 10, 12^ and $k_{x}$ and $k_{z}$ are the wave numbers in *x* and *z* directions, respectively. Therefore, both the one-dimensional sinusoidal and square-checkerboard modes were considered as special cases of the relation above in that for the perfectly equi-biaxial compression, $k_{x}=k_{z}=\left( 1/\sqrt{2} \right)k_{cr}$, and for uniaxial compression, $k_{x}=$ $k_{cr}$ and $k_{z}=0$. Therefore, the critical stress defined in Eq. (3) also holds for the square-checkerboard mode under equi-biaxial loading. The critical strain for the square-checkerboard mode, $\left( e_{cr} \right)_{CB}$, is apparently derived^9, 10, 12^ by dividing $\sigma_{cr}$ by the biaxial modulus of the film, ${E_{f}}/\left( 1-\upsilon_{f} \right)$,

$$\left( e_{cr} \right)_{CB}=\frac{\sigma_{cr}}{\left[ {E_{f}}/\left( 1-\upsilon_{f} \right) \right]}=\left[ \frac{1}{4\left( 1+\upsilon_{f} \right)} \right]\left[ \frac{3\left( 1-\upsilon_{f}^{2} \right)\bar{E}_{s}}{E_{f}} \right]^{2/3}. (6)$$

In addition, the amplitude of the surface wrinkles, $A$, can be written in a general form as

$$A=\Psi\left( \frac{e}{e_{cr}}-1 \right)^{1/2}, (7)$$

where the parameter $\Psi$ is a function of Poisson’s ratio and thickness of the film layer, with $\Psi$ =$t_{f}$ for the sinusoidal 1D mode and $\Psi$ =$t_{f}\cdot\sqrt{8/{[\left( 3-\upsilon_{f} \right)\left( 1+\upsilon_{f} \right)}]}$ for the square-checkerboard mode ^9, 12^, and $e/e_{cr}$ is the applied compressive strain normalized by the critical value at the onset of primary bifurcation.

It should be mentioned that, an alternative wavelength expression different from Eq. (5) exists in the literature, for the square-checkerboard pattern created under equi-biaxial loading. Audoly et al. ^11^ proposed an analytical form based on the linear stability analysis, with $\left( \lambda_{cr} \right)_{Cb}=\left( \lambda_{cr} \right)_{1D}$. This solution was based on superposition of two perpendicular 1D modes, and is apparently inconsistent with the predictions from other works^9, 10, 12, 13^. From our previous simulation study^23^, the predicted wavelength for the square-checkerboard mode is in line with Eq. (5).

In the literature the primary bifurcation mode caused by perfectly equi-biaxial compression has been regarded as “unusual,” as it was theoretically predicted that “multiplicity of periodic modes” associated with $\sigma_{cr}$, defined in Eq. (3), exists due to their identical energy state. Chen and Hutchinson^10, 24^ reported that, under equi-biaxial compression at the onset of wrinkling, any deformation mode that satisfy $\sqrt{k_{x}^{2}+k_{z}^{2}}=k_{cr}$ can possibly form (which include both the 1D and square-checkerboard modes). This claim was also followed by the analytical approach proposed by Song et al.^12^ where they apparently postulated that both the 1D mode and square-checkerboard mode can form under equi-biaxial compression, with Eq. (6) applicable to both. A different view was proposed by Audoly et al.^11^; they analytically showed that under equi-biaxial loading, the initial instability mode is square-checkerboard which has the lowest energy. It was also shown that the hexagonal pattern is yet another analytically possible mode under equi-biaxial loading, but energetically less favorable than the square-checkerboard. Moreover, under non-equi-biaxial loading, the 1D mode was predicted to have the lowest energy at the initial buckling threshold and thus be the dominant initial pattern. The “multiplicity of periodic modes” reported by Chen and Hutchinson^10, 24^ was later updated by Cai et al.^9^, where the 1D mode, square-checkerboard, hexagonal mode, and newly introduced triangular mode were postulated to be the possible solutions under equi-biaxial compression. Based on their upper-bound analysis on a flat elastic film-substrate, the square-checkerboard mode has the lowest energy; the hexagonal and triangular modes were found to have exactly the same energy but higher than that of the square-checkerboard, and the 1D mode was the least favorable mode of all under the equi-biaxial condition. In addition, the 1D mode was shown to be the predominant mode under non-equi-biaxial loading, in consistency with the predictions of Audoly et al.^11^. Cai et al.^9^ also showed that linear combinations of hexagonal and triangular modes lead to various possible solutions that have exactly the same energy as the hexagonal and triangular modes.

Although the most energetically favorable mode under equi-biaxial loading is the square-checkerboard^9, 11^, other checkerboard patterns (including the hexagonal and triangular modes) have been frequently observed in actual experiments^9^. A true square-checkerboard pattern can only form under pre-defined conditions using special fabrication methods^25, 26^. As a consequence, researchers have speculated the possible causes of the discrepancy. A preexisting curvature of the film surface has been considered as a possible cause to initiate hexagonal-mode wrinkles^9, 14^. Nonlinear elastic property of the substrate^9^ and unequal substrate elastic moduli in tension and compression have also been proposed as the origin for the hexagon-based checkerboard patterns^13^.

There are other well recognized surface instability patterns such as herringbone (or zigzag) and labyrinth, which emerge after the primary modes when the compressive stress is well beyond the first critical point. Uncertainties exist in the literature regarding their evolution and necessary loading condition in the post-instability regime. Chen and Hutchinson^10, 24^ analytically showed that under high overstresses (stresses well above the critical value $\sigma_{cr}$), the herringbone pattern has the minimum energy state compared to the 1D and square-checkerboard modes. There are apparent ambiguities in the literature about the necessary loading condition for triggering the herringbone mode. Some analytical studies included the herringbone pattern in the group of surface wrinkles that can develop under perfect equi-biaxial loading^9, 10, 12, 24, 27^; while others^11, 13, 15^ followed the analytical study of Audoly et al.^11^ and treated the herringbone pattern as a post-1D cylindrical pattern (secondary bifurcation) which evolves under non-equi-biaxial loading^11^. (Note that non-equi-biaxial loading is also frequently termed “anisotropic loading” in the literature).

The wave parameters of the herringbone pattern have been studied analytically. On the basis of their upper-bound analysis, Cai et al.^9^ predicted that herringbone arises as a bifurcation of the square checkerboard mode under equi-biaxial loading, and it has the lowest energy when $\sigma/{\sigma_{cr}}\geq1.476$with $\upsilon_{f}=1/3$ and when $\sigma/{\sigma_{cr}}\geq1.595$with $\upsilon_{f}=1/2$. (They also showed that square-checkerboard has the lowest energy when $1{\leq\sigma}/{\sigma_{cr}}\leq1.476$ with $\upsilon_{f}=1/3$). Some studies used two wavelengths (along with the corresponding wave numbers) to delineate the uniform herringbone pattern: a short wavelength ($\lambda_{1}={2\pi}/{k_{1}}$), and a long wavelength ($\lambda_{2}={2\pi}/{k_{2}}$). In non-equi-biaxial loading, $\lambda_{1}$ will form parallel to the loading direction with a higher stress magnitude, and $\lambda_{2}$ is associated with the lateral undulation in the direction of lower stress magnitude which is perpendicular to the $\lambda_{1}$ waves (note that generally $\lambda_{2}>\lambda_{1}$). According to Chen and Hutchinson^10^ and the works following their study^12, 15, 27^, at the minimum energy state where herringbone is the dominant mode, the small wavelength roughly equals to the wavelength of the 1D mode at the onset of instability, $\lambda_{1}=\left( \lambda_{cr} \right)_{1D}$ defined in Eq. (1), and the jog angle is 45° (angle associated with the $\lambda_{2}$ zigzags). Following the claim that the herringbone pattern is a secondary bifurcation of the 1D mode under anisotropic loading, the presumption of $\lambda_{1}=\left( \lambda_{cr} \right)_{1D}$ was also stated in Audoly et al.^11^ and followed by other subsequent studies^9, 13^. Nevertheless, no reliable closed-form solutions have been introduced to capture $\lambda_{1}$, $\lambda_{2}$ and the jog angle as well as their evolution. The von Karman partial differential equation, however, can be simplified under various assumptions, and thus simpler ordinary differential equations for the herringbone pattern can be obtained and solved numerically^9, 12, 13^. As for the labyrinth pattern, it was also claimed that the relation $\lambda_{1}=\left( \lambda_{cr} \right)_{1D}$ remains unchanged^9, 13, 15^; similar to herringbone, this pattern has not been analytically studied.

**Sequence of wrinkle patterns**

Various scenarios of surface pattern development have been reported and they are summarized here. Under perfect equi-biaxial loading, two different evolution paths have been analytically predicted so far: (1) from an initial flat surface to square-checkerboard and then to herringbone^9, 10, 24^, and (2) from an initial flat surface to square-checkerboard (or hexagonal) and then directly to labyrinth^13, 15, 28^. On the other hand, under non-equi-biaxial compression, the analytically or numerically predicted paths include: (1) from an initial flat surface to 1D mode and then to herringbone^11^, (2) from an initial flat surface to 1D mode and then to herringbone, and finally to labyrinth^13, 15, 28^, (3) from an initial flat surface to hexagonal mode and then to labyrinth (for substrates displaying elastic asymmetry in tension and compression)^13^, and (4) from an initial curved surface to 1D mode and then to hexagonal mode, and finally to labyrinth^14^.

It is evident from the different predictions that, even with a flat film-substrate system with a simple elastic behavior, a unified theme for the evolution of wrinkle patterns is lacking. Furthermore, how transitions occur from one post-instability mode to another is not at all clear. The effects of loading biaxiality are also in need of investigating. The work presented in the main body of this paper addresses all these issues and demonstrates the modeling capabilities using the embedded imperfection approach.

# References

1. Allen HG. Analysis and design of structural sandwich panels. *Analysis and design of structural sandwich panels*, 1 edn. Pergamon Press Ltd.: Headinton Hill Hall, Oxford, London, 1969, pp 76-97.

2. Koiter W, Pignataro M. Buckling Structures, ed. by B. Budiansky. Springer-Verlag; 1976.

3. Biot MA, Drucker DC. Mechanics of Incremental Deformation. *Journal of Applied Mechanics* 1965, **32**(4)**:** 957-958.

4. Biot MA. Surface instability of rubber in compression. *Applied Scientific Research, Section A* 1963, **12**(2)**:** 168-182.

5. Chung JY, Nolte AJ, Stafford CM. Surface wrinkling: a versatile platform for measuring thin‐film properties. *Advanced Materials* 2011, **23**(3)**:** 349-368.

6. Volynskii A, Bazhenov S, Lebedeva O, Bakeev N. Mechanical buckling instability of thin coatings deposited on soft polymer substrates. *Journal of materials science* 2000, **35**(3)**:** 547-554.

7. Mei H, Landis CM, Huang R. Concomitant wrinkling and buckle-delamination of elastic thin films on compliant substrates. *Mechanics of Materials* 2011, **43**(11)**:** 627-642.

8. Groenewold J. Wrinkling of plates coupled with soft elastic media. *Physica A: Statistical Mechanics and its Applications* 2001, **298**(1-2)**:** 32-45.

9. Cai S, Breid D, Crosby AJ, Suo Z, Hutchinson JW. Periodic patterns and energy states of buckled films on compliant substrates. *Journal of the Mechanics and Physics of Solids* 2011, **59**(5)**:** 1094-1114.

10. Chen X, Hutchinson JW. Herringbone buckling patterns of compressed thin films on compliant substrates. *J Appl Mech* 2004, **71**(5)**:** 597-603.

11. Audoly B, Boudaoud A. Buckling of a stiff film bound to a compliant substrate—Part I:: Formulation, linear stability of cylindrical patterns, secondary bifurcations. *Journal of the Mechanics and Physics of Solids* 2008, **56**(7)**:** 2401-2421.

12. Song J, Jiang H, Choi W, Khang D-Y, Huang Y, Rogers JA. An analytical study of two-dimensional buckling of thin films on compliant substrates. *Journal of Applied Physics* 2008, **103**(1)**:** 014303.

13. Huang X, Li B, Hong W, Cao Y-P, Feng X-Q. Effects of tension–compression asymmetry on the surface wrinkling of film–substrate systems. *Journal of the Mechanics and Physics of Solids* 2016, **94:** 88-104.

14. Zhao Y, Zhu H, Jiang C, Cao Y, Feng X-Q. Wrinkling pattern evolution on curved surfaces. *Journal of the Mechanics and Physics of Solids* 2020, **135:** 103798.

15. Huang Z, Hong W, Suo Z. Nonlinear analyses of wrinkles in a film bonded to a compliant substrate. *Journal of the Mechanics and Physics of Solids* 2005, **53**(9)**:** 2101-2118.

16. Chen Y-c, Yang S, Wheeler L. Surface instability of elastic half-spaces by using the energy method. *Proceedings of the Royal Society A: Mathematical, Physical and Engineering Sciences* 2018, **474**(2213)**:** 20170854.

17. Ciarlet PG. A justification of the von Kármán equations. *Archive for Rational Mechanics and Analysis* 1980, **73**(4)**:** 349-389.

18. Ciarlet PG. *Plates and junctions in elastic multi-structures: an asymptotic analysis*, vol. 14. Masson, 1990.

19. Nikravesh S, Ryu D, Shen Y-L. Instabilities of Thin Films on a Compliant Substrate: Direct Numerical Simulations from Surface Wrinkling to Global Buckling. *Scientific Reports* 2020, **10**(1)**:** 1-19.

20. Nikravesh S, Ryu D, Shen Y-L. Surface Instability of Composite Thin Films on Compliant Substrates: Direct Simulation Approach. *Frontiers in Materials* 2019, **6:** 214.

21. Ma Y, Xue Y, Jang K-I, Feng X, Rogers JA, Huang Y. Wrinkling of a stiff thin film bonded to a pre-strained, compliant substrate with finite thickness. *Proceedings of the Royal Society A: Mathematical, Physical and Engineering Sciences* 2016, **472**(2192)**:** 20160339.

22. Wang C, Zhang S, Nie S, Su Y, Chen W, Song J. Buckling of a stiff thin film on a bi-layer compliant substrate of finite thickness. *International Journal of Solids and Structures* 2020, **188:** 133-140.

23. Nikravesh S, Ryu D, Shen Y-L. Instability driven surface patterns: Insights from direct three-dimensional finite element simulations. *Extreme Mechanics Letters* 2020**:** 100779.

24. Chen X, Hutchinson JW. A family of herringbone patterns in thin films. *Scripta materialia* 2004, **50**(6)**:** 797-801.

25. Yoo PJ, Suh KY, Park SY, Lee HH. Physical self‐assembly of microstructures by anisotropic buckling. *Advanced materials* 2002, **14**(19)**:** 1383-1387.

26. Li M, Qin H, Liu J, Liu Y. Mechanism of three-dimensional surface wrinkle manipulation on a compliant substrate. *Journal of Applied Mechanics* 2018, **85**(7).

27. Choi WM, Song J, Khang D-Y, Jiang H, Huang YY, Rogers JA. Biaxially stretchable “wavy” silicon nanomembranes. *Nano Letters* 2007, **7**(6)**:** 1655-1663.

28. Huang Z, Hong W, Suo Z. Evolution of wrinkles in hard films on soft substrates. *Physical Review E* 2004, **70**(3)**:** 030601.
